# Fennel (*Foeniculum vulgare*) on management of menopausal symptoms: A protocol for systematic review of randomized controlled trials

**DOI:** 10.1097/MD.0000000000010223

**Published:** 2018-03-30

**Authors:** Hye Won Lee, Hyun-Ja Lim, Ji Hee Jun, Hyun-Suk Lim, Myeong Soo Lee

**Affiliations:** a Korea Institute of Oriental Medicine, Daejeon; b Department of Nursing, Chodang University, Muan; c Department of Nursing, Howon University, Kunsan; d Department of Korean Medicine Life Science, Korea University of Science & Technology, Daejeon, Republic of Korea.

**Keywords:** fennel, menopausal symptom, menopause, systematic review

## Abstract

**Background:**

Fennel (*Foeniculum vulgare*) is often used in women's health care to treat dysmenorrhea, increase the milk supply, and address symptoms of menopause. The object of this review is to evaluate the current evidence on the efficacy of fennel for the management menopausal symptoms.

**Methods and analyses:**

Thirteen databases will be searched from their inception to the present. These include PubMed, AMED, EMBASE, the Cochrane Library, six Korean medical databases (Korean Studies Information Service System, DBPIA, the Korean Institute of Science and Technology Information, the Research Information Service System, KoreaMed, and the Korean National Assembly Library), and 3 Chinese databases (the China National Knowledge Infrastructure Database [CNKI], the Chongqing VIP Chinese Science and Technology Periodical Database [VIP], and Wanfang Database). Study selection, data extraction, and assessments will be performed independently by 2 researchers. The risk of bias will be assessed using the Cochrane risk of bias tool.

**Ethics and dissemination:**

Ethical approval is not required, given that this protocol is for a systematic review only. The review will be published in a peer-reviewed journal and disseminated both electronically and in print. The review will be updated to inform and guide healthcare practice and policy.

**Trial registration number:**

PROSPERO 2018 CRD42018085698.

## 1 Introduction

A recent systematic review showed that >50% of menopausal women often use complementary and alternative medicine (CAM) to manage the symptoms of menopause.^[1]^ Many menopausal women use CAM because of the possible long-term risk of hormone replacement therapy; they see CAM as a safer approach.^[2–4]^ The most popular form of CAM is herbal medicine.^[1]^ Several herbal medicines were mentioned in the recent review, including black cohosh, St John's Wort, ginseng, and dangui, but evidence of their clinical efficacy remains limited.^[5]^

Fennel (*Foeniculum vugare*) has been used as a herb for medicinal purposes and as a food; its seed and root are popular seasonings.^[6]^ It is reported as effective for reducing dysfunction in the gastrointestinal tract, increasing milk supply, exerting estrogenic activity, and managing dysmenorrhea.^[6,7]^ Several studies have demonstrated that fennel is effective for relaxing smooth muscles, improving memory, and increasing antioxidant effects.^[6,8,9]^ Recently, clinical trials have claimed fennel's influence on the management of postmenopausal symptoms.^[10,11]^ However, no systematic or meta-analysis has been conducted to date for fennel as a means of managing the symptoms of menopause. The objective of this systematic review is to determine whether fennel is effective for managing these symptoms.

## 2 Methods

### 2.1 Study registration

This protocol review has been registered on PROSPERO 2018 CRD42018085698 (http://www.crd.york.ac.uk/PROSPERO/display_record.php?ID=CRD42018085698).

### 2.2 Criteria for considering studies for this review

#### 2.2.1 Types of studies

Prospective randomized controlled trials (RCTs) will be included, regardless of publication language. Quasi RCTs will be excluded. We will also exclude observational, cohort, case report, case series, non-RCT, animal, and experimental studies.

#### 2.2.2 Types of participants

Menopausal women will be included. We will exclude studies with breast cancer patients, patients with endometriosis, those who are immunocompromised, or who take multiple medications. Menopausal symptoms include hot flashes, psychological health, insomnia, and so on.

#### 2.2.3 Types of interventions and controls

All types of fennel (*F. vugare*) will be included for consideration, such as pills, extracts, supplements, oil used for massage or inhalation, capsules, or in raw form. Trials in which fennel formed part of a complex herbal medicine will be excluded. Trials that combine fennel with conventional therapies will be included, if the control group received the same conventional therapies as the experimental group. We will include those trials that compare fennel with a placebo, conventional therapies, or usual cares. Trials comparing fennel with other types of complementary therapies will be excluded.

#### 2.2.4 Types of outcome measures

##### 2.2.4.1 Primary outcomes

1. Menopausal symptoms, as measured by the Kupperman index or other questionnaires for scoring menopausal symptoms
2. Response rate

##### 2.2.4.2 Secondary outcomes

1. Quality of life
2. Adverse events

### 2.3 Search method for identifying the studies

#### 2.3.1 Electronic searches

The following databases were searched from their inception to the present: PubMed, AMED, EMBASE, the Cochrane Library, Korean medical databases (the Korean Studies Information Service System, DBPIA, the Korean Institute of Science and Technology Information, the Research Information Service System, KoreaMed, and the Korean National Assembly Library), the China National Knowledge Infrastructure Database (CNKI), the Chongqing VIP Chinese Science and Technology Periodical Database (VIP), and Wanfang Database. Articles identified through reference lists of included studies and relevant systematic reviews were also considered for inclusion.

#### 2.3.2 Search strategy

The search terms used will be “(fennel OR *foeniculum vulgare*) AND (menopause$ OR climact$ OR perimenopaus$ OR peri-menopaus$ OR post menopause$ OR post-menopaus$ OR hot flash$ OR hot- flash$ OR hot flush$ OR hot-flush$).”

### 2.4 Data collection, extraction, and assessment

#### 2.4.1 Selection of studies

Two reviewers (HWL and JHJ) will independently screen the titles and abstracts for searched studies and make study selections. They will record their decisions according to predefined criteria. Another reviewer (MSL) will resolve disagreements regarding study selection. Study selection will be documented and summarized via a Preferred Reporting Items for Systematic Reviews and Meta-Analysis (PRISMA) flow diagram.^[12]^

#### 2.4.2 Data extraction

Two reviewers (HWL and HJL) will read the studies and independently extract data using a standard data extraction form. The form will be composed of participants, intervention group treatment, control group treatment, outcomes, and results. Disagreements will be resolved by another reviewer (MSL). We will use Grading of Recommendations Assessment, Development and Evaluation (GRADE) software (GRADEpro GDT, https://gradepro.org/) to judge the quality of evidence for data from Cochrane Systematic Reviews to create a Summary of Findings table. In addition, the details of the treatment regimens will be summarized in a table. When reported data are insufficient or unclear, a researcher will contact the first author or corresponding author by e-mail or telephone to request missing or clarified data.

#### 2.4.3 Assessment of risk of bias

The risk of bias will be assessed using the Cochrane risk of bias criteria: random sequence generation, allocation concealment, blinding of participants and personnel, blinding of outcome assessment, incomplete outcome data, selective outcome reporting, and other sources of bias (we will evaluate baseline imbalances).^[13]^ This review will use L, U, and H as a key for these judgments, where “low” (L) indicates a low risk of bias, “unclear” (U) indicates that the risk of bias is uncertain, and “high” (H) indicates a high risk of bias. Disagreements will be resolved by discussions among all authors. The risk of bias assessment for the included studies is summarized in a table, and the results and implications will be discussed from a critical point of view.

### 2.5 Data synthesis

All statistical analyses will be conducted using the Cochrane collaboration's software program Review Manager (RevMan), v 5.3 for Windows (Copenhagen, The Nordic Cochrane Centre). Differences between the intervention and placebo control groups will be assessed. In the analysis of clinical efficacy, categorical data will be assessed in terms of risk ratios, and continuous data will be assessed in terms of mean difference (MD). Categorical and continuous variables will be expressed as efficacy values with 95% confidence intervals (CIs). In cases of outcome variables with different scales, the standardized MD will be used instead of the weighted MD. We will use a fixed model if there is no evidence of heterogeneity; if there is, we will apply the random effect model. If a meta-analysis is possible, we will use the *I*^2^ statistic for quantifying inconsistencies across the included studies.^[14]^ A resulting 50% cutoff point will represent substantial heterogeneity. If heterogeneity is observed, we will conduct subgroup analyses. Subgroup analyses will be conducted according to the type of fennel, dose, and treatment duration. Where appropriate, sensitivity analyses will be performed to evaluate the robustness of the meta-analysis results.

If >10 studies are available, we will complete a funnel plot for publication bias and small study effects using Egger method.^[15]^ Funnel plot asymmetry is certainly not the same as publication bias. We will attempt to distinguish the possible reasons for any asymmetry; therefore, we consider the possible poor methodological quality and true heterogeneity of all studies.

### 2.6 Ethics and dissemination

Ethical approval is not required, as this protocol is for a systematic review only. The findings of this review will be disseminated widely through peer-reviewed publications and conference presentations.

## 3 Discussion

This protocol for a systematic review will provide a detailed summary of the current state of evidence regarding the effectiveness of fennel in treating the symptoms of menopausal women. The review will be useful to patients and healthcare providers in determining the appropriate role of fennel in the management of menopausal symptoms.

## Author contributions

**Conceptualization:** H.W. Lee, M.S. Lee.

**Data curation:** H-J. Lim, H-S. Lim.

**Formal analysis:** J.H. Jun.

**Funding acquisition:** H.W. Lee, M.S. Lee.

**Methodology:** M.S. Lee.

**Software:** J.H. Jun.

**Supervision:** M.S. Lee.

**Validation:** H-J. Lim, H-S. Lim, J.H. Jun, M.S. Lee.

**Writing – original draft:** H.W. Lee, M.S. Lee.

**Writing – review & editing:** H-J. Lim, H-S. Lim, J.H. Jun.
